# Effects of *Lonomia obliqua* Venom on Vascular Smooth Muscle Cells: Contribution of NADPH Oxidase-Derived Reactive Oxygen Species

**DOI:** 10.3390/toxins9110360

**Published:** 2017-11-07

**Authors:** João Alfredo Moraes, Genilson Rodrigues, Vany Nascimento-Silva, Mariana Renovato-Martins, Markus Berger, Jorge Almeida Guimarães, Christina Barja-Fidalgo

**Affiliations:** 1 Laboratório de Biologia RedOx, Instituto de Ciências Biomédicas, Universidade Federal do Rio de Janeiro, Rio de Janeiro CEP 21941-902, Brazil; jmoraesbr@yahoo.com; 2 Laboratório de Farmacologia Celular e Molecular, Departamento de Biologia Celular, Universidade do Estado do Rio de Janeiro, Rio de Janeiro CEP 20550-030, Brazil; biogenilson@yahoo.com.br (G.R.); vanysilva@hotmail.com (V.N.-S.); m_renovatomartins@yahoo.com.br (M.R.-M.); 3 Laboratório de Bioquímica Farmacológica, Centro de Pesquisa Experimental, Hospital de Clínicas de Porto Alegre (CPE/HCPA/UFRGS), Porto Alegre CEP 90035-903, Brazil; mbergeroliveira@gmail.com (M.B.); jguimaraes14@gmail.com (J.A.G.); 4 Centro de Biotecnologia, Universidade Federal do Rio Grande do Sul (UFRGS), Porto Alegre CEP 951501-970, Brazil

**Keywords:** *Lonomia obliqua* venom, vascular smooth muscle cells, reactive oxygen species

## Abstract

Envenomation caused by human contact with the caterpillar Lonomia is characterized by deleterious effects on coagulation and patency of blood vessels. The cellular effects induced by *Lonomia obliqua* venom highlights its capacity to activate endothelial cells, leading to a proinflammatory phenotype. Having more knowledge about the mechanisms involved in envenomation may contribute to better treatment. We aimed to evaluate the effects of *Lonomia obliqua* caterpillar bristle extract (LOCBE) on vascular smooth muscle cells (VSMC). We observed that LOCBE induced VSMC migration, which was preceded by alterations in actin cytoskeleton dynamics and Focal Adhesion Kinase activation. LOCBE also induced Extracellular Signal-Regulated Kinase (ERK) phosphorylation in VSMC, and the inhibition of this pathway impaired cell proliferation. Stimulation of VSMC with LOCBE triggered reactive oxygen species (ROS) production through the activation of NADPH oxidase. The rapid increase in these ROS further induced mitochondrial ROS production, however only NADPH oxidase-derived ROS were involved in ERK activation in VSMC. We that demonstrated the chemotactic and proliferative effects of LOCBE on VSMC were dependent on ROS production, mainly through NADPH oxidase. Together, the data show that *Lonomia obliqua* venom can interact with and activate VSMC. These effects rely on ROS production, suggesting new potential targets for treatment against vascular damage during envenomation.

## 1. Introduction

Lonomism is the envenomation caused by caterpillars belonging to the Lonomia genus. There are two species of Lonomia that cause harm to humans: *Lonomia obliqua* and *Lonomia achelous* [1]. Envenomation caused by *L. achelous* has been reported in Venezuela since 1967 [2] and this caterpillar is found in Venezuela, Bolivia, Colombia, Ecuador, French Guyana, Suriname, and North Brazil. *Lonomia obliqua* species are mainly found in South Brazil and neighboring countries, and the species has also spread to the southeast of the country, contributing to an increase in the number of reported accidents [1]. Envenomation by *L. obliqua* occurs when a person accidentally comes into contact with a colony containing up to hundreds of caterpillars camouflaged on the tree trunks. Due to this contact, the animals are usually crushed and the venom, as a mixture of venom and hemolymph, is injected subcutaneously by the broken bristles. Due to the usually high number of caterpillars in a single colony, a considerable amount of venom can be injected into the victim leading to serious cases of envenomation [3].

The initial clinical manifestations of *L. obliqua* envenomation are similar to contact dermatitis, including pain and a burning sensation at the site of contact. However, as the course of envenomation progresses, more severe symptoms appear, such as bleeding from skin and mucous membranes, and generalized hemorrhage within a period up to 72 h after contact [4,5,6]. Severe cases can evolve into acute kidney injury, which is the main cause of death by envenomation [7]. The venom can directly modulate the victim’s hemostatic system by the proteolytic activation of coagulation, fibrinolytic, and kinin cascades, generating high concentrations of intravascular thrombin, plasmin, and kallikrein [8,9,10]. Together, the factors released during the envenomation act on the vascular system, increasing vascular permeability, and inducing hypotension, as well as nociceptive and edematogenic responses [11,12,13,14].

We showed that *L. obliqua* venom can induce the expression of several pro-inflammatory and procoagulant mediators in endothelial cells and fibroblasts, which led these cells to acquire a pro-inflammatory profile, contributing to the disturbances in the vascular system [9,14]. In an experimental in vivo model of envenomation in rats, significant amounts of venom could be detected in the vascular smooth muscle cells (VSMC) of glomerular capillaries, which can be associated with increasing tissue factor expression and intraglomerular fibrin deposition [15].

Together with endothelium, the VSMC are considered a key component in vascular inflammatory processes. Under pro-inflammatory stimuli, the VSMC undergo oxidative stress, due to the exacerbated production of reactive oxygen species (ROS) derived from the NADPH oxidase system or from mitochondrial respiratory chain uncoupling. Once activated, VSMC can acquire a migratory and proliferative phenotype, which may lead to atheromatous plaque development, restenosis, and cardiovascular alterations [16].

We hypothesized that *L. obliqua* venom could directly activate and modulate the physiology of VSMC. Our results showed that *L. obliqua* venom could change the VSMC functionality by triggering signaling pathways that are dependent on NADPH oxidase-derived and mitochondrial-derived ROS, and Extracellular Signal-Regulated Kinase-2 (ERK-2) activation, as well as inducing VSMC activation, migration, and proliferation.

## 2. Results

### 2.1. Effect of LOCBE on VSMC Migration

**Figure 1:**
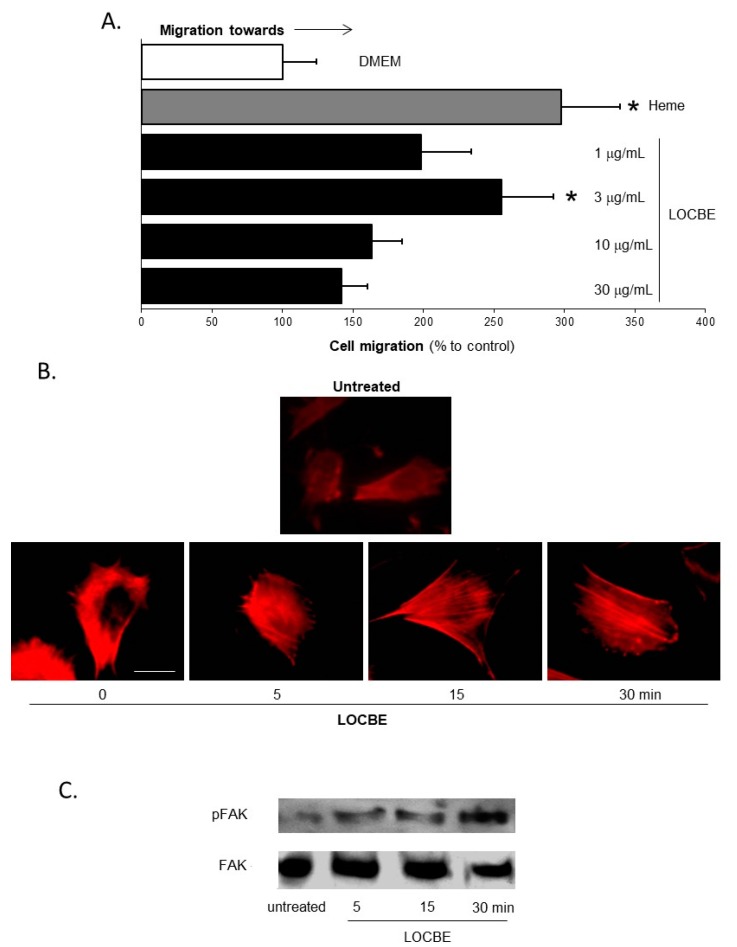
Effect of *Lonomia obliqua* caterpillar bristle extract (LOCBE) on vascular smooth muscle cell migration. (**A**) Cultured vascular smooth muscle cells (VSMC) were placed in a modified Boyden chamber and chemotaxis was induced by LOCBE (1–30 µg/mL) or heme (10 µM). Membranes were stained and migrating cells were counted via light microscopy. Results are representative of five different experiments. Data are means ± Standard Deviations of the Means (SDM). * denotes *p* < 0.05 vs. untreated VSMC. (**B**) VSMC were cultured on glass coverslips overnight, and the next day cells were incubated with LOCBE (3 µg/mL) for 5–30 min at 37 °C. As described in Section 2, actin filaments were stained with Tetramethylrhodamine (TRITC)-phalloidin. Cells were imaged by fluorescence microscopy and the images shown are representative of the five different fields observed in one experiment. The results represent three different experiments. (**C**) Cultured VSMC were incubated with or without LOCBE (3 µg/mL) for 5, 15, and 30 min at 37 °C. Whole-cell lysates were immunoblotted with anti-phospho Focal Adhesion Kinase (pFAK) polyclonal (upper inset) and with anti-Focal Adhesion Kinase (FAK) monoclonal antibodies (lower inset).

To evaluate the effect of LOCBE on VSMC, we first investigated if LOBCE has chemotactic properties. Here, we studied the migratory effect of LOCBE in modified Boyden chambers after four hours’ incubation. LOCBE appears to be a potent chemotactic agent, given the characteristic bell-shape curve at concentrations of 1, 3, 10, and 30 µg/mL, with an increase at 3 µg/mL and a decrease at high concentrations. At 3 µg/mL, LOCBE induced an effect similar to heme, used as the positive control (Figure 1A).

The reorganization of the actin cytoskeleton is a key process required for cell migration. Since LOCBE was able to induce VSMC migration, we investigated the changes in the dynamics of the actin cytoskeleton. In cells stained with rhodamine-phalloidin, LOCBE (3 µg/mL) induced VSMC actin cytoskeleton rearrangement, just after five minutes of stimulation (Figure 1B). Cells assumed a typical migratory shape, polarized by lammelipodium formation and stress fibers crossing the cell body. These changes were maintained for 30 min, the last time observations were recorded. Furthermore, several studies have shown that Focal Adhesion Kinase (FAK) is a pivotal molecule in cell migration. Once activated, FAK forms focal adhesions, and cell adhesion and coordinated movement occur. Accordingly, we demonstrated that LOCBE (3 µg/mL) induced FAK phosphorylation at five minutes and had the highest phosphorylation at 30 min (Figure 1C). This continuous FAK activation was directly related to the cell migration that occurred during the four hours after treatment (Figure 1A).

### 2.2. Effect of LOCBE on VSMC Proliferation

**Figure 2:**
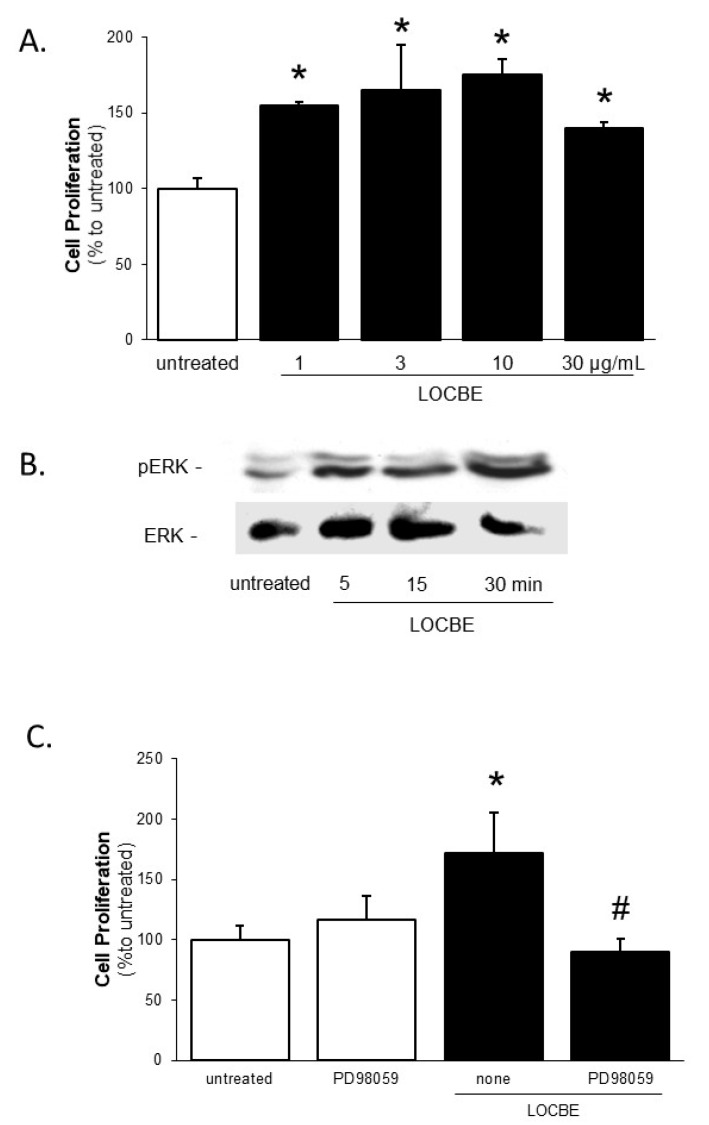
(**A**) Effect of LOCBE on vascular smooth muscle cell proliferation. (**B**) VSMC were incubated with or without LOCBE (1–30 µg/mL) for 48 h at 37 °C. Cell proliferation was determined using the thymidine incorporation assay. The results represent four different experiments. (**C**) Cultured VSMC were incubated with or without LOCBE (3 µg/mL) for 5, 15, and 30 min, at 37 °C. Whole-cell lysates were immunoblotted with anti-phospho Extracellular Signal-Regulated Kinase (pERK) polyclonal (upper inset) and with anti-Extracellular Signal-Regulated Kinase-2 (ERK-2) polyclonal antibody (lower inset). The result represents three different experiments. Cultured VSMC were incubated with or without LOCBE (3 µg/mL) for 48 h at 37 °C and, when indicated, were pretreated with Extracellular Signal-Regulated Kinase (ERK) inhibitor PD98059 (10 µM). Cell proliferation was determined using the thymidine incorporation assay. The results represent four different experiments. Data are means ± SDM; * denotes *p* < 0.05 vs. untreated VSMC; # denotes *p* < 0.05 vs. VSMC treated with LOCBE.

**Figure 3:**
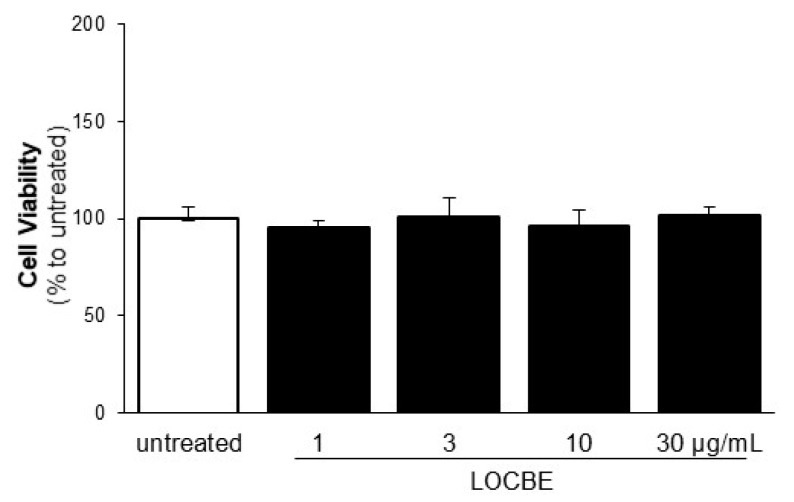
Effect of LOCBE on vascular smooth muscle cell viability. Cultured VSMC were incubated with or without LOCBE (1–30 µg/mL) for 48 h at 37 °C. Cell viability was determined in optical microscopy through trypan blue exclusion count. The results are representative of four different experiments. Data are means ± SDM. The results are representative of three different experiments.

The next stage of our study investigated cell proliferation, another crucial event during VSMC activation. We observed that LOCBE showed a significant pro-proliferative effect on VSMC at all concentrations tested (1–30 µg/mL) 48 h after treatment (Figure 2A). Notably, LOCBE (1, 3, 10, and 30 µg/mL) did not have any effect on VSMC viability (Figure 3).

To understand the mechanism of the LOCBE effect on cell proliferation, we focused on Extracellular Signal-Regulated Kinase (ERK), an important member of Mitogen Activated Protein Kinase, which modulates the proliferative activity of different cell types [17]. The LOCBE (3 µg/mL) treatment of VSMC induced ERK-2 phosphorylation in a time-dependent manner, peaking at 30 min (Figure 2B). To investigate the role of this signaling pathway, VSMC were pre-treated with PD98059, an ERK inhibitor, and subjected to LOCBE treatment. Inhibition of the ERK pathway impaired the LOCBE (3 µg/mL) effect on VSMC proliferation (Figure 2C), demonstrating the involvement of ERK-2 in this process.

### 2.3. Effect of LOCBE on VSMC ROS Production

**Figure 4:**
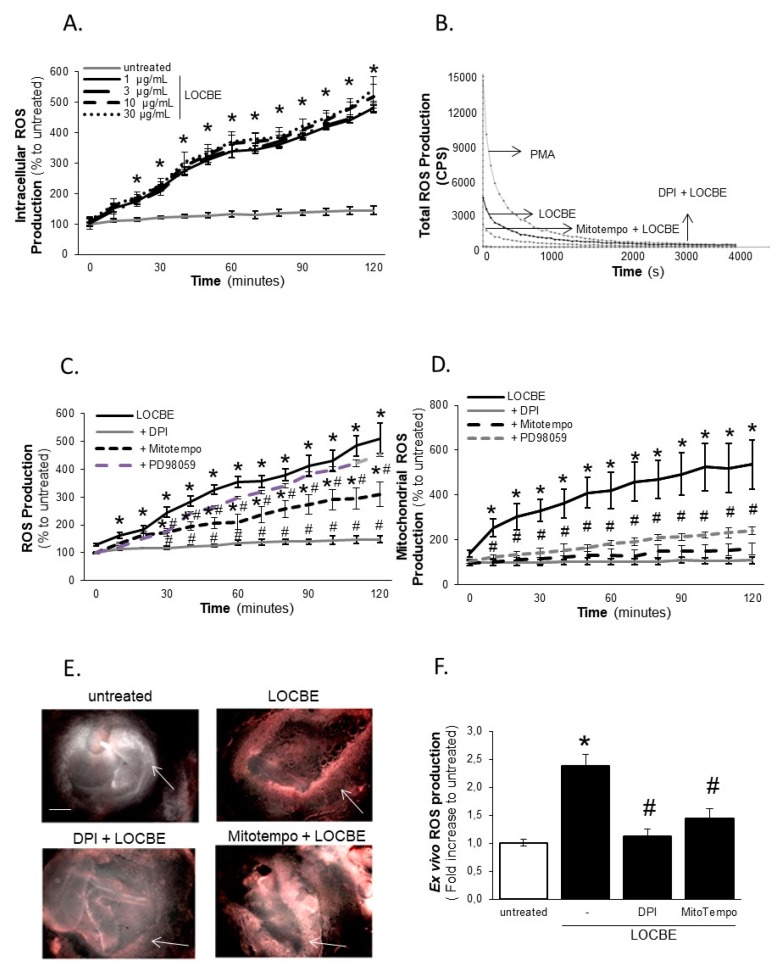
Effect of LOCBE on vascular smooth muscle cell reactive oxygen species production. (**A**,**C**) Intracellular ROS analysis cells previously loaded with a DCF probe (10 µM). (**B**) Total ROS analysis cells co-treated with luminol probe and Phorbol 12-myristate 13-acetate (PMA) were used as a positive control. (**D**) Mitochondrial ROS analysis cells previously loaded with MitoSox probe (10 µM). Each experiment was performed in triplicate. (**E**) Slides of thoracic aorta were pretreated with diphenyleneiodonium chloride (DPI) (10 µM) or Mitotempo (10 µM) for 15 min, then cells were washed three times with phosphate-buffered saline (PBS) and were loaded with dihydroethidium (DHE) (5 μM). Then, the cells were stimulated with LOCBE (3 µg/mL) for 30 min at 37 °C, protected from light and washed three times with PBS. Slides were analyzed under fluorescence microscopy. (**F**) The images were analyzed and quantified using Photoshop software. The results represent three different experiments. Data are means ± SDM; * denotes *p* < 0.05 vs. untreated VSMC; # denotes *p* < 0.05 vs. VSMC treated with LOCBE.

**Figure 5:**
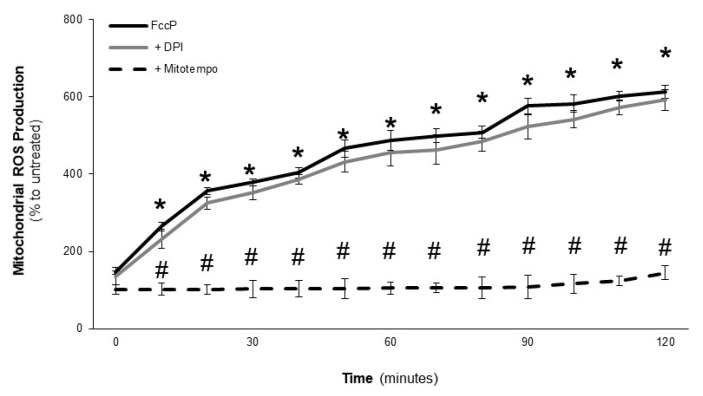
Effect of DPI on vascular smooth muscle cell mitochondrial-derived reactive oxygen species production. Cultured VSMC were pre-treated or not with DPI or Mitotempo (10 µM) for 15 min, then cells were incubated with or without FccP (5 µM) for 2 h at 37 °C. Cells were previously loaded with MitoSox probe and ROS production was performed in multiplate reader Envision. The results are representative of three different experiments. Data are means ± SDM; * denotes *p* < 0.05 vs. untreated VSMC; # denotes *p* < 0.05 vs. VSMC treated with FccP.

Increases in ROS production, which are mainly intracellular, have been shown to modulate different VSMC functions [18,19]. Thus, we investigated whether LOCBE could induce oxidative stress in VSMC. Figure 4A shows that LOCBE, in all concentrations tested (1–30 µg/mL), induced a rapid (10 min) and potent intracellular ROS production, as determined with a CM-H_2_DCFDA (DCF) probe, which was sustained until 120 min (the end of the study time period). When we investigated total ROS production with a luminol probe, we observed that LOCBE at 3 µg/mL was able to induce ROS production in an NADPH oxidase system-dependent manner, once diphenyleneiodonium chloride (DPI), a general NADPH oxidase inhibitor, abrogated its effect (Figure 4B). Additionally, we observed that the effect of LOCBE (3 µg/mL) on ROS production, in terms of total and intracellular ROS, relied partially on the mitochondrial complex, once it was partially inhibited by Mitotempo (Figure 4B,C). Since we observed the role of mitochondria in the LOCBE effect on ROS production, we focused on mitochondria-derived ROS production using a MitoSox probe. Figure 4D shows that LOCBE (3 µg/mL) was able to induce mitochondrial-derived ROS, and its production was dependent on the NADPH oxidase system. As expected, the mitochondrial-derived ROS inhibitor, Mitotempo, totally inhibited the LOCBE effect (Figure 4D). In the same analysis, we observed that the LOCBE effect on mitochondrial ROS production was partially dependent on ERK activation, since PD98059 partially inhibited this effect (Figure 4D). PD98059 did not affect the LOCBE effect on intracellular ROS production (Figure 4C). Some studies have found that DPI can also act as an inhibitor of mitochondrial ROS production, however, in this work, we observed that DPI was not able to inhibit mitochondrial ROS production induced by the electron transport chain uncoupler FccP (Figure 5).

The LOCBE effects on ROS production were also investigated ex vivo in rat aorta slices with a dihydroethidium (DHE) probe. The results observed are shown in Figure 3E, and quantified in Figure 4F, showing that LOCBE (3 µg/mL) induced ROS production predominantly in VSMC obtained from the aortic media layer. The effect of LOCBE on aorta tissue also relied on NADPH oxidase activity, being blocked by DPI treatment, and partially derived from mitochondria, and it was partially inhibited by Mitotempo (Figure 4E,F).

### 2.4. LOCBE-Induced ROS Production Modulates VSMC Functions

**Figure 6:**
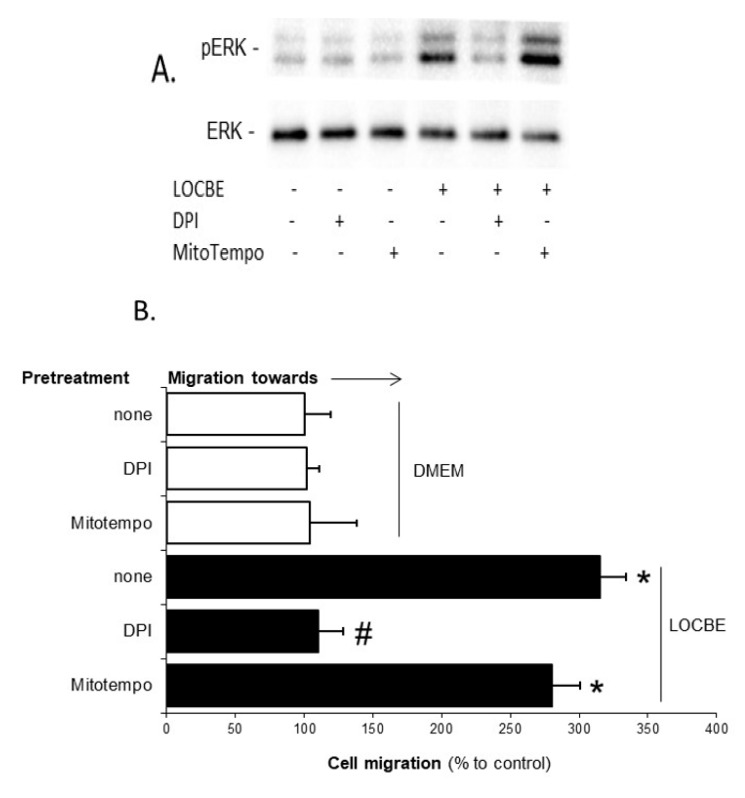

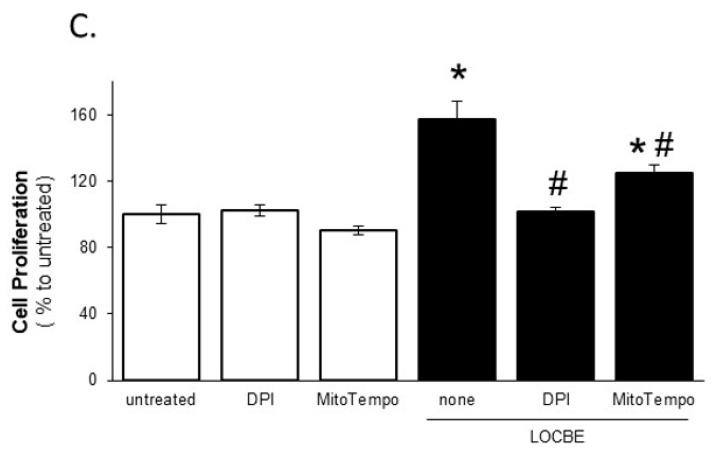
LOCBE-induced ROS production modulates effects on VSMC. (**A**) Cultured VSMC were incubated with or without LOCBE (3 µg/mL) for 30 min at 37 °C and when indicated, cells were pretreated with DPI (10 µM) or Mitotempo (10 µM). Whole-cell lysates were immunoblotted with anti-phospho ERK polyclonal (upper inset) and with anti-ERK-2 polyclonal antibody (lower inset). The results represent three experiments. (**B**) Cultured VSMC were placed in a modified Boyden chamber and chemotaxis was assessed to LOCBE (3 µg/mL) and when indicated, cells were pretreated with DPI (10 µM) or Mitotempo (10 µM). Membranes were stained and migrating cells were counted using light microscopy. Results represent three different experiments. (**C**) Cultured VSMC were incubated with or without LOCBE (3 µg/mL) for 48 h at 37 °C and when indicated, were pretreated with DPI (10 µM) or Mitotempo (10 µM). Cell proliferation was determined using the thymidine incorporation assay. The results represent three different experiments. Data are means ± SDM; * denotes *p* < 0.05 vs. untreated VSMC; # denotes *p* < 0.05 vs. VSMC treated with LOCBE.

Finally, we investigated the effects on VSMC due to the involvement of ROS derived from the NADPH oxidase system or from the mitochondria in LOCBE. Figure 6A shows that only DPI inhibited LOCBE (3 µg/mL), affecting ERK activation. Furthermore, we observed that only DPI blocked cell migration toward LOCBE (3 µg/mL) (Figure 6B), indicating that NADPH oxidase-derived ROS is crucial for these processes. When we investigated LOCBE-induced (3 µg/mL) cell proliferation, we observed that its effect was totally dependent on the NADPH oxidase system and partially dependent on the mitochondrial-derived ROS (Figure 6C).

## 3. Discussion

*L. obliqua* venom is composed of several active constituents, including serine proteases, lipocalins, phospholipases A2, lectins, protease inhibitors, and biologically active peptides. Those molecules may act directly, or contribute to the generation of endogenous mediators (as kinins, chemokines, and cytokines) [20,21], and some of them are potential inducers of vascular injury. Systemically, the main signs of vascular alterations during envenomation are an increase in microvascular permeability, hypotension, and a decrease in glomerular filtration rate [15,22]. We showed that *L. obliqua* venom was able to directly activate endothelial cells, changing their phenotype to a pro-inflammatory profile, increasing endothelial permeability and the expression of adhesion molecules (E-selectin and VCAM-1) and pro-inflammatory cytokines regulated by the NF-κB pathway [14]. Complementing the data, we have now demonstrated that vascular alterations induced by *L. obliqua* venom might also be due to its direct effects on VSMC, inducing oxidative stress, and the modification of the functionality of vascular cells.

In general, the process of vascular injury is accompanied by endothelial cell lesion, extracellular matrix exposure, thrombus formation and leukocyte adhesion followed by VSMC activation, which can accumulate at the site of lesion [23]. The VSMC dysfunction is characterized by an exacerbation of cell migration and proliferation, events that are amplified by the release of inflammatory mediators [18,23,24,25,26]. Although during the envenomation, the venom is primarily injected into the victim’s subcutaneous tissue, significant amounts of toxins are able to reach the vascular system, including the VSMC [11,15]. Then, we raised the hypothesis that *L. obliqua* venom could lead to VSMC dysfunction. LOCBE was shown to be a potent activator of VSMC, able to induce cell chemotaxis, proliferation, and ROS production in almost all concentrations tested from 1 to 30 µg/mL.

Cellular migration is a well-ordered process, dependent on specific alterations in actin cytoskeleton dynamics [27]. The challenges of VSMC stimulated with LOCBE were increased actin polymerization, promoted actin stress-fiber formation, and induced cell polarization. The rearrangement of the actin cytoskeleton in LOCBE-stimulated VSMC was accompanied by FAK phosphorylation, a key event during the signaling mechano-transduction in many cells [28]. Upon activation, FAK acted as a platform to recruit and activate other kinases, including members of the MAPK family, which can modulate cell functions and fate [17]. LOCBE, in parallel to FAK activation, induced ERK-2 phosphorylation. The downstream activation of the ERK-2 pathway seemed to be directly associated with LOCBE-induced VSMC proliferation.

Notably, the exacerbated activation, migration, and proliferation of VSMC could effectively contribute to venom-induced pathology. The accumulation of these cells, which favors restenosis, together with intravascular coagulation, can reduce blood flow and tissue oxygen supply causing ischemia. Ischemia is a common event observed mainly in the lungs and kidneys after envenomation [29]. Moreover, venom-induced intravascular hemolysis could be enhanced during this process as free Heme, released during the hemolytic process, which is a strong pro-oxidant and pro-inflammatory molecule that also contributes to VSMC proliferation [26].

In this study, novel data show LOCBE as a potent inducer of ROS production by VSMC, and that these ROS were responsible for the majority of the exacerbated effects of LOCBE in VSMC. We demonstrated that LOCBE induced ROS production from NADPH oxidase, which in turn triggerd ROS production from mitochondria. Moreover, NADPH oxidase-derived ROS mediated the LOCBE effect on VSMC migration and proliferation, whereas the mitochondrial-derived ROS only partially mediated the LOCBE effect on VSMC proliferation.

We also showed that only NAPDH oxidase-derived ROS was able to activate ERK, the main pathway responsible for cell proliferation [17]. We suggest that part of the effect of ERK on cell proliferation was mediated by mitochondrial-derived ROS, once we observed that ERK was involved in mitochondrial ROS production and that this ROS was partially involved in cell proliferation.

## 4. Conclusions

**Figure 7:**
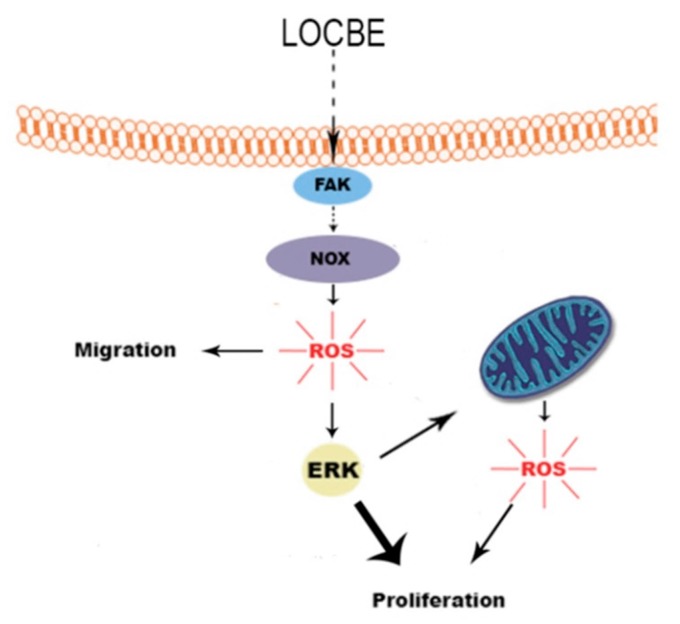
LOCBE effects on vascular smooth muscle cells. LOCBE activated FAK and, consequently, actin cytoskeleton rearrangement and cell migration. LOCBE also induced ROS generation from the NADPH oxidase system, which modulated ERK activation and cell migration and proliferation. NADPH oxidase-derived ROS–ERK activation also induced ROS production from mitochondria, which is partially involved in VSMC proliferation.

As summarized in Figure 7, LOCBE was able to trigger FAK activation and, consequently, actin cytoskeleton rearrangement and cell migration. Once activated by LOCBE, VSMC was able to generate ROS from the NADPH oxidase system, which modulated ERK activation and cell migration and proliferation.

## 5. Materials and Methods

### 5.1. Reagents

Heme, HEPES, trypsin, EDTA, bovine serum albumin (BSA), PMSF, benzamidine, leupeptin, soybean trypsin inhibitor, and Mitotempo were obtained from Sigma-Aldrich (St. Louis, MO, USA). Diphenyleneiodonium chloride (DPI) and PD98059 were from Calbiochem (Darmstadt, Germany). Dulbecco’s modified Eagle’s medium (DMEM) and fetal calf serum (FCS) were from GIBCO-BRL (Carlsbad, CA, USA). Antibodies anti-ERK-2, anti-ERK, anti-FAK, and anti-pFAK were purchased from Santa Cruz Biotechnology (Santa Cruz, CA, USA). CM-H_2_DCFDA (DCF), Dihydroethidium (DHE), Luminol, and MitoSox were from Life Technologies (Carlsbad, CA, USA). The enhanced chemiluminescence (ECL) system and BCA protein assay kit were obtained from Pierce Biotechnology (Rockford, IL, USA).

### 5.2. Venom

*L. obliqua* caterpillars were provided by the Centro de Informações Toxicológicas (CIT), Porto Alegre, Rio Grande do Sul, Brazil. The specimens used in the present study were collected in the cities of Bom Princípio and Progresso, both located at Rio Grande do Sul, Brazil. *L. obliqua* venom was obtained from bristles cut at the base of each scoli, macerated in cold phosphate-buffered saline (PBS), at pH 7.4, as previously described [9,10,20,30]. This *Lonomia obliqua* Caterpillar Bristle Extract (LOCBE) had its protein concentration determined using a BCA assay kit (Pierce, Rockford, IL, USA). Venom samples were aliquoted and stored at −80 °C prior to use.

### 5.3. Cell Culture

A7r5 cells obtained from rat thoracic aorta were from the American Type Culture Collection (Rockville, MD, USA). The cells were cultured in DMEM medium containing 10% FCS, 50 U/mL penicillin, and 100 µg/mL streptomycin. The cultures were incubated at 37 °C in a 5% carbon dioxide (CO_2_) air atmosphere. The cells were passaged at confluence following dissociation with 0.1%/0.01% trypsin/EDTA and then seeded into new culture flasks for a maximum of 12 passages.

### 5.4. Cell Migration

Aliquots of 100,000 cells in DMEM were seeded per well on 48 wells in a modified Boyden chamber mounted with a 10 µm pore-polycarbonate membrane (Neuroprobe, Gaithersburg, MD, USA). Some samples were pre-incubated with either DPI (10 µM) or Mitotempo (10 µM) for 15 min before addition to the upper chamber (50 µL). LOCBE (1–30 µg/mL) or Heme (10 µM; positive control) were added to the lower chamber (27 µL). After incubation at 37 °C in a 5% CO_2_ atmosphere for 4 h, cells that migrated to the lower membrane surface were fixed and stained by Giemsa. Cells adherent to the bottom of the membranes were counted under optical microscopy (400× magnification). For each well, five randomly chosen visual fields were counted blindly, and the mean value was used as a measure of VSMC migration. In each experiment, twelve wells containing medium without LOCBE in the lower chamber were used as a negative control.

### 5.5. Cell Proliferation

Aliquots of 10,000 VSMC were seeded in 96-well plates overnight in a DMEM medium containing 10% FCS. The cells were washed three times with PBS and the medium was replaced by DMEM containing 1% FCS for 1 h. When indicated, cells were pretreated with DPI (10 µM), Mitotempo (10 µM), or PD98059 (10 µM) for 15 min. Then, LOCBE (1–30 µg/mL) was added and the cells were incubated for 48 h at 37 °C in a 5% CO_2_ air atmosphere. Twenty-four hours before the end of incubation, thymidine [H^3^] was added to all wells. After incubation, the cells were washed two times with PBS and fixated with TCA 10% for 20 min. Then the cells were washed two times with PBS and lysed with NaOH 0.2 N. The cellular content was transferred to a dense filter and remained overnight to dry. The radioactivity that remained on the filters was detected in a scintillation counter and results were expressed in counts per minute (CPM).

### 5.6. Reactive Oxygen Species Production

VSMC (5 × 10^3^ cells/well) were seeded in 96-well black plates (DCF and MitoSox) or white plates (luminol) overnight in DMEM medium containing 10% FBS. The cells were washed three times with PBS and the medium was replaced by DMEM containing 1% FCS for 1 h. For intracellular ROS detection, VSMC were loaded with DCF (10 µM) or MitoSox (10 µM) for 1 h and then washed to remove free probe. For total ROS detection, VSMC were co-incubated with luminol (50 µM). Cells were pretreated with DPI (10 µM), Mitotempo (10 µM), or PD98059 (10 µM) for 15 min, and then incubated with LOCBE (1–30 µg/mL) for 2 h at 37 °C in a 5% CO_2_ air atmosphere. CM-H2DCFDA fluorescence was monitored at excitation and emission wavelengths of 495 and 530 nm, respectively, and MitoSox was monitored at excitation and emission wavelengths of 490 and 515 nm, respectively. Luminol-emitted luminescences were measured at 5-second intervals throughout a 1 h period. Fluorescence and chemiluminescence were quantified using the EnVision™ multilabel plate reader (Perkin-Elmer, Waltham, MA, USA).

### 5.7. Ex Vivo ROS Production Assay

Rats were anesthetized with ketamine and xylazine (90 and 15 mg/kg, respectively, i.p.) and perfused with saline (NaCl 0.9%). The thoracic aorta was dissected and aorta slides were pretreated with DPI (10 µM) or Mitotempo (10 µM) for 15 min, then cells were washed 3 times with PBS and were loaded with DHE (5 μM). The cells were then stimulated with LOCBE (3 µg/mL) for 30 min at 37 °C, protected from light. The slides were analyzed under fluorescence microscopy (Olympus BX41 microscope, Olympus, Tokyo, Japan). The images were analyzed using Photoshop software (Adobe Systems, San Jose, CA, USA). At least 3 images of each aorta slice were taken (*n* = 3 per group).

This study was performed in strict accordance with the recommendations in the Guide for the Care and Use of Laboratory Animals of the National Institutes of Health, USA. The protocol was approved by the Committee on the Ethics of Animal Experimental of Federal University of Rio de Janeiro (Permit number: CEUA/080/2016).

### 5.8. Immunofluorescence Microscopy

VSMC were plated (5 × 10^4^ cells) on glass coverslips overnight in a DMEM medium containing 10% FCS and grown on glass coverslips at 37 °C in a 5% CO_2_ air atmosphere. The cells were washed three times with PBS, the medium was replaced by serum-free medium and after 30 min, and the cells were stimulated with LOCBE (3 µg/mL) for different lengths of time. The monolayers were washed twice with PBS and the cells were fixated with 4% paraformaldehyde/4% sucrose in PBS for 20 min and blocked with 5% BSA in PBS for 30 min. Then, VSMC were washed three times with PBS and incubated for 2 h with phalloidin-TRITC (1:1000) at room temperature. Coverslips were mounted on a slide with the use of a 20 mM *N*-propylgalate and 80% glycerol solution in PBS before examination under a microscope (model BX40 Olympus, Tokyo, Japan) equipped for epifluorescence. The images were analyzed using Photoshop software (Adobe Systems, San Jose, CA, USA).

### 5.9. Cellular Extract

VSMC were cultured (10^6^ cells) on 6-well microtiter plates with DMEM 10% FCS for 24 h. Then the cells were washed with PBS and incubated with serum-free DMEM for 1 h. Groups that received pre-treatment with DPI (10 µM) or Mitotempo (10 µM) were incubated for 15 min, and after, cells were incubated for different lengths of time with LOCBE (1–30 µg/mL) at 37 °C in a 5% CO_2_ air atmosphere. Then, VSMC were lysed in lysis buffer (50 mM HEPES, pH 6.4, 1 mM MgCl_2_, 10 mM EDTA, 1% Triton X-100, 1 µg/mL DNase, 0.5 µg/mL Rnase, 1 mM PMSF, 1 mM benzamidine, 1 µg/mL leupeptin, and 1 µM/mL soybean trypsin inhibitor).

### 5.10. Western Blot Analysis

Total protein content in the cell extracts was determined using the BCA method. Cell lysates were denatured in sample buffer (50 mM Tris·HCl, pH 6.8, 1% SDS, 5% 2-ME, 10% glycerol, and 0.001% bromophenol blue) and heated in a boiling water bath for 3 min. Samples (30 µg total protein) were resolved by 10% SDS-PAGE and proteins were transferred to polyvinylidinedifluoride membranes. Molecular weight standards were run in parallel to estimate molecular weights. Membranes were blocked with Tween-PBS (T-PBS) (PBS, 0.01% Tween 20) containing 1% BSA and probed with primary antibody (1:1000) overnight at 4 °C. The membranes were rinsed with T-PBS and incubated for 1 h at room temperature with Horseradish Peroxidase-conjugated secondary antibody (1:10,000). Immunoreactive proteins were visualized by ECL detection. The bands were quantified by densitometry with the use of Photoshop software (Adobe Systems, San Jose, CA, USA).

### 5.11. Statistical Analysis

Statistical significance was assessed by ANOVA, followed by Bonferroni’s *t*-test, and *p* < 0.05 was taken as statistically significant.
